# Protection against West Nile Virus Infection in Mice after Inoculation with Type I Interferon-Inducing RNA Transcripts

**DOI:** 10.1371/journal.pone.0049494

**Published:** 2012-11-14

**Authors:** Miguel Rodríguez-Pulido, Miguel A. Martín-Acebes, Estela Escribano-Romero, Ana-Belén Blázquez, Francisco Sobrino, Belén Borrego, Margarita Sáiz, Juan-Carlos Saiz

**Affiliations:** 1 Centro de Biología Molecular Severo Ochoa, Consejo Superior de Investigaciones Científicas-Universidad Autónoma de Madrid (CSIC-UAM), Madrid, Spain; 2 Departamento de Biotecnología, Instituto Nacional de Investigación y Tecnología Agraria y Alimentaria (INIA), Madrid, Spain; 3 Centro de Investigación en Sanidad Animal, Instituto Nacional de Investigación y Tecnología Agraria y Alimentaria (CISA-INIA), Madrid, Spain; University of Texas Medical Branch, United States of America

## Abstract

West Nile virus (WNV) is a neurovirulent single stranded RNA mosquito-borne flavivirus, whose main natural hosts are birds, but it also infects humans and horses. Nowadays, no human vaccine is commercially available and clinical treatment is only supportive. Recently, it has been shown that RNA transcripts, mimicking structural domains in the non-coding regions (NCRs) of the foot-and mouth disease virus (FMDV) induce a potent IFN response and antiviral activity in transfected cultured cells, and also reduced mice susceptibility to FMDV. By using different transcripts combinations, administration schedules, and infecting routes and doses, we have demonstrated that these FMDV RNA transcripts protect suckling and adult mice against lethal challenge with WNV. The protective activity induced by the transcripts was systemic and dependent on the infection route and dose. These results confirm the antiviral potential of these synthetic RNAs for fighting viruses of different families relevant for human and animal health.

## Introduction

West Nile virus (WNV), a flavivirus (*Flaviviridae* family), cycles between mosquitoes and birds, but also infects a broad range of mammals, including humans and horses [1], [2]. After its first description in 1937 [3], WNV had been associated with sporadic outbreaks of meningoencephalitis in Africa, Europe, and the Middle East until 1990's. Since then, an increase in the number, frequency and severity of West Nile disease (WND) cases in horses and humans has been documented in Europe [1], [2]. In 1999 WNV emerged for the first time in the US [4] causing thousands of infections among humans, horses, and birds. Although WNV infections in humans and horses are mainly subclinical, clinically apparent infections range from a febrile illness (West Nile fever) to a neuroinvasive disease associated with a relatively high mortality [1], [2], [5]. Currently, there is no vaccine approved for use in humans and clinical treatment is only supportive [6]. Therefore, search for antiviral compounds is a pivotal key for anti-WNV prophylaxis.

The innate immune response is a first line defense against invading pathogens that depends on several sensors and signaling pathways. The detection of viral products as pathogen-associated molecular patterns (PAMPs), including single and double-stranded RNAs, initiates a signaling cascade that leads to rapid antiviral response, including the secretion of IFN-α and IFN-β, which have well known antiviral, antiproliferative and immunomodulatory functions [7].

WNV is highly sensitive to interferon. Administration of IFN to a limited number of WNV-infected patients helped to reduce disease complications in some of them, although in others failed to do so [8]–[10]. As many other viruses, WNV has developed different strategies to block the action IFN and thus, to evade the host antiviral activity of IFN-stimulating genes, ISGs [6], [11]. Different reports indicate that WNV non-structural proteins contribute to control IFN-α/β signaling by different ways [6]. On the other hand, genetic polymorphism of the IFN-inducible 2′5′-oligoadenylate synthetases (OAS) has been involved in the host innate resistance to WNV infection in horses [12], humans [13] and mice [14]. RNA motifs in the 3′ non-coding regions (NCRs) of the hepatits C virus (*Flaviviridae* family) have been described as IFN inducers when transfected into cultured cells and mice [15], [16], but a role of subgenomic RNAs from the 3′ NCR of WNV in evading IFN response has also been recently reported [17]. In addition, a genetic deficiency on the chemokine receptor CCR5 has been associated with enhanced mortality in mice [18] and humans [19]. Some IFN regulatory factors (IRFs) have been implicated in systemic production of IFN-α, while other do not have any appreciable effect [20] but, their role in protection against WNV infection *in vivo* remains elusive [6], [21]. Pretreatment of cells with IFN inhibits flavivirus infection, but its effect is markedly attenuated once viral replication has begun [22], as non-structural viral proteins antagonize IFN effects [6]. Pretreatment of rodents with IFN-α also reduced viral loads and mortality [23].

Recently, the ability of transcripts mimicking structural domains in the 5′ and 3′ NCR of foot-and mouth disease virus (FMDV, *Picornaviridae* family) genome to trigger IFN-β activity in cultured cells has been reported [24]. Even more, when inoculated into suckling mice, they were also able to trigger the innate immune response and to reduce the susceptibility of the animals to FMDV infection [24], [25]. The level of the protective *in vivo* response was dependent on the specific RNA and the dose administered and was cross-protective against different FMDV serotypes [25].

Here, we have tested the capability of the 5′NCR S and IRES transcripts, proved to induce the highest protective effect in mice against FMDV, to protect suckling and adult mice against challenge with WNV. Our results confirm the wide prophylactic potential of these molecules for viral control strategies.

## Materials and Methods

### Ethics Statement

All animals were handled in strict accordance with the guidelines of the European Community 86/609/CEE at the biosafety animal facilities of the Centro de Investigación en Sanidad Animal Of the Instituto Nacional de Investigación Agraraia y Alimentaria (CISA-INIA) The protocols were approved by the Committee on Ethics of Animal Experimentation of INIA (permit numbers 2011–15 and 2011–35). Animals were monitored twice daily and received water and food *ad libitum*. All surgical manipulations were performed under anesthesia and efforts were made to minimize suffering.

### Virus and RNAs

West Nile virus NY-99 strain was propagated and titrated on Vero cells as described [26]–[27]. RNA transcripts corresponding to the S and IRES fragments (403 and 470 nucelotide-long, respectively) of the 5′ NCR of FMDV O1K and CS-8 genome, respectively, were generated by *in vitro* transcription as described [24].

#### Mice

Litters of 7 to 17 newborn (4–7 days-old) Swiss mice were intraperitoneally (i.p.) inoculated with 100 µg of the corresponding transcripts (either S or IRES), or poly (I:C) (Sigma), in a final volume of 100 µl in PBS as described [24]. In the case of RNA transcripts, 20 µg of lipofectin (Invitrogen), which has been shown to have no influence in the protection induced by the transcripts [25], was also added. After 24 h of RNA inoculation, mice were infected with WNV, either i.p. (10^2^ or 10^7^ PFU/mouse), or intracraneally, i.c. (10 or 10^2^ PFU/mouse). As a control, groups of mice were infected with the same amount of virus that had been pre-incubated for 1 h at 37°C with a mice neutralizing polyclonal anti-WNV sera pool (n-sera) [28]. Likewise, two groups of mice were i.c. or i.p. inoculated with PBS 24 h before infection, and two additional good practice control groups were inoculated with PBS by the same routes, but they were not challenged with WNV. Additionally, litters of newborns were i.p. inoculated as above with 100 µg of S, IRES, or an equimolar mixture of both transcripts, and i.p. challenged with WNV (10^2^ PFU/mouse) 1 day before or 5 days after inoculation of the RNAs. The mixture was not tested in the case of i.p. infection 5 days after treatment.

On the other hand, groups of 18 adult (6–8 weeks-old) Swiss mice were i.p. inoculated with 200 µg of S or IRES transcripts 24 h or 48 h before, or 48 h after being i.p. infected with WNV (10^5^ PFU/mouse). A group of mice was pretreated with 200 µg of poly (I:C) and a second one with PBS alone 24 h before infection. Mice were bled 4, 8, and 24 h after treatment with the transcripts. On day 3, 4 and 5 after infection, 2 randomly selected mice per group were anesthetized, bled, euthanized, and their brains collected and processed [26] for viral and immunological determinations.

During the experiments mice were monitored twice daily and received water and food *ad libitum*. Those mice showing clear signs of disease were anesthetized and euthanized, as were all surviving animals at the end of the experiment (4 weeks after infection). All experiments with infectious virus were conducted in biosafety level 3 facilities.

### Immunological and viral assays

Antibodies against WNV were measured by a validated ELISA using WNV baculovirus expressed recombinant protein E as antigen [29]. The positive cut off value was assigned using a positive/negative (P/N) ratio ≥2, calculated by dividing the mean absorbance of the test serum reacted on viral antigen by the absorbance of the negative control serum on viral antigen. Virus neutralization test (VNT) was performed, using serial serum dilutions, in susceptible Vero cells _as reported_ [30]. Titers were calculated as the reciprocal of the serum dilution that completely inhibited cytophatic effect at 1∶20 or higher dilution.

Viral RNA was extracted from processed brains using a NucleoSpin viral RNA isolation kit (Macherey-Nagel) and quantified by real-time RT-PCR [26], [31]. For RNA quantification a standard curve was generated with previously titrated WNV (10^6^–10^−1^ PFU), and samples were considered negative when Ct≥35, equivalent to 10^2^ PFU/gram of tissue [26], [32].

The levels of IFN-α in pools of sera from adult mice inoculated with the different transcripts, the poly (I:C), or with PBS alone were tested by ELISA using a commercial kit (PBL InterferonSource). The limit of detection of IFN-α in serum samples in the conditions assayed was 25 µg/ml, as determined in previous assays (unpublished results). Antiviral activity of sera (1∶1 dilution) was also analyzed by a cytophatic effect inhibition test as described [24], [33], and expressed as the highest dilution of sera (log_2_) able to suppress vesicular stomatitis virus (VSV)-induced cytophatic effect on L-929 cells in 50% of the wells.

### Statistical analyses

Kaplan-Meier survival curves were analysed by a logrank test using GraphPad PRISM v.2.01 (GraphPad Software). Statistically significant differences were considered at p<0.05. The median survival time (MST) was calculated for every time and group of inoculated mice.

## Results

**Figure 1:**
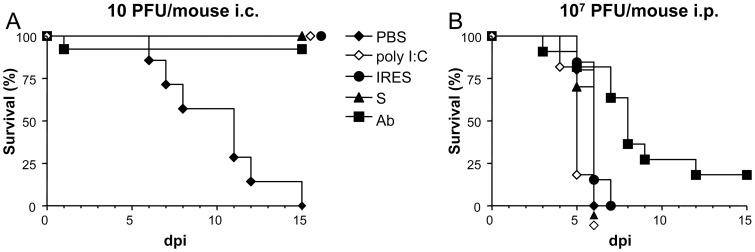
Survival rates of suckling mice treated with the different transcripts 24 h before infection with WNV. Mice were i.p. inoculated with the corresponding transcripts (either S or IRES), or with poly (I:C), or PBS alone 24 h before being i.c. infected with 10 PFU/mouse (A), or i.p. with 10^7^ PFU/mouse (B), as described in Materials and Methods. As a control, a group (Ab) of mice was infected with the same amount of virus previously incubated with a neutralizing polyclonal anti-WNV sera pool.

All suckling mice inoculated 24 h before infection, either with the S, IRES, or poly (I:C), a synthetic double stranded RNA homologue that has been shown to induce anti-viral activity in suckling mice [23]–[25], [34], and almost all (92.3%) of those in which the virus was previously incubated with n-sera [28], survived to intracraneal (i.c.) infection with the lowest dose of WNV tested, 10 PFU/mouse (Fig. 1A). As expected, all mice in the PBS-inoculated control group died of WND.

On the contrary, all suckling mice inoculated with S, IRES, poly (I:C), or PBS 24 h before being intraperitoneally (i.p.) infected with a very high dose (10^7^ PFU) of WNV died 4 to 7 days later with median survival time (MST) of 6 days for the S, IRES, and PBS, and 5 days for the poly (I:C) (Fig. 1B). A high mortality (81.2%) was also recorded in the control group in which the virus had been incubated with n-sera before the infection, as only two animals survived, although in this case the MST was higher (8, range 5–15 days, logrank test p<0.0029). No mortality was recorded in the good practice groups, in which mice were i.c. or i.p. inoculated with PBS but not infected (data not shown).

**Figure 2:**
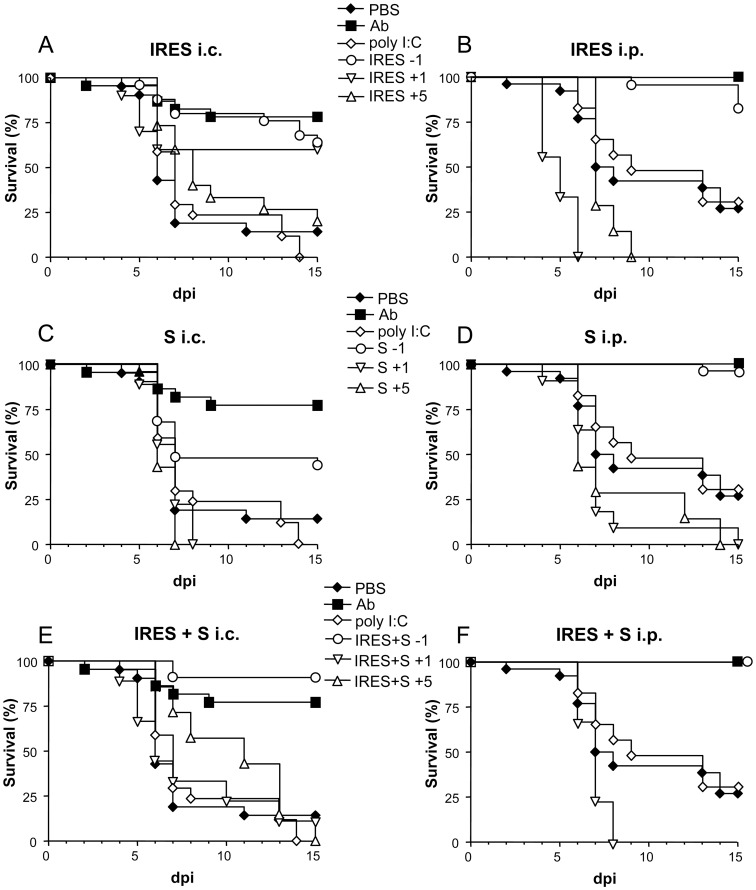
Survival rates of WNV infected suckling mice treated with the different transcripts at various time intervals. Mice were i.p. inoculated with the corresponding transcripts, either the IRES (A and B), the S fragment (C and D), or an equimolar mixture of both, IRES+S (E and F) 1 day before (−1), or 1 (+1) or 5 (+5) days after i.c. (A, C and E) or i.p. (B, D and F) infection with 10^2^ PFU/mouse of WNV, as described in Materials and Methods. Survival rates of mice inoculated with poly (I:C), PBS alone, or virus that had been previously incubated with a neutralizing polyclonal anti-WNV sera pool (Ab), 24 h before infection are shown in each panel for comparison.

When newborn mice were i.c. infected with 100 PFU (Fig. 2 A, C and E), a statistically significant high survival rate was observed among those inoculated 24 h before with the RNA transcripts, either the IRES (64%, p≤0.0001), the S fragment (44%, p≤0.0208), or an equimolar mixture of both (90.9%, p≤0.0001), in comparison with the group of PBS-inoculated mice (14.3%). Similar results were recorded in the control group, n-sera, (81.8% of survivors, p≤0.0001). In contrast, none of the 17 mice inoculated with poly (I:C) survived to the infection.

Even superior protection rates were observed when the virus was inoculated i.p. 24 h after treatment (Fig. 2 B, D and F), since highest survival rates were recorded among mice pretreated with the IRES (82.6%, p≤0.0001), the S fragment (96.4%, p≤0.0001), or the mixture of both (100%, p≤0.0007), as well as in the control n-sera group (100%, p≤0.0001), when compared with those observed in mice treated with poly (I:C) (33.3% survival, p≤0.6272) or with PBS alone (28% survival).

The protective capacity of the S and IRES RNA transcripts, and of an equimolar mixture of both, when administered 1 or 5 days after infection, was further compared with that observed when they were inoculated 24 h before infection (Fig. 2). Some protection (60% and 20%) was only observed when the IRES transcript was administered 1 or 5 days after i.c. infection (Fig. 2A), although these figures did not reach statistical significance (p≤0.089 and 0.405, respectively) when compared with the control group. No protection was recorded in any other group in which the transcripts were inoculated after infection, except for a single mouse (11.2%) that survived to i.c infection 24 h after treatment with the equimolar mixture of both RNAs (Fig. 2E). In most cases, MST values were lower in mice treated 1 or 5 days after infection than in those treated 24 h before. Likewise, these values also tended to be lower in i.c. than in i.p. infected mice.

**Figure 3:**
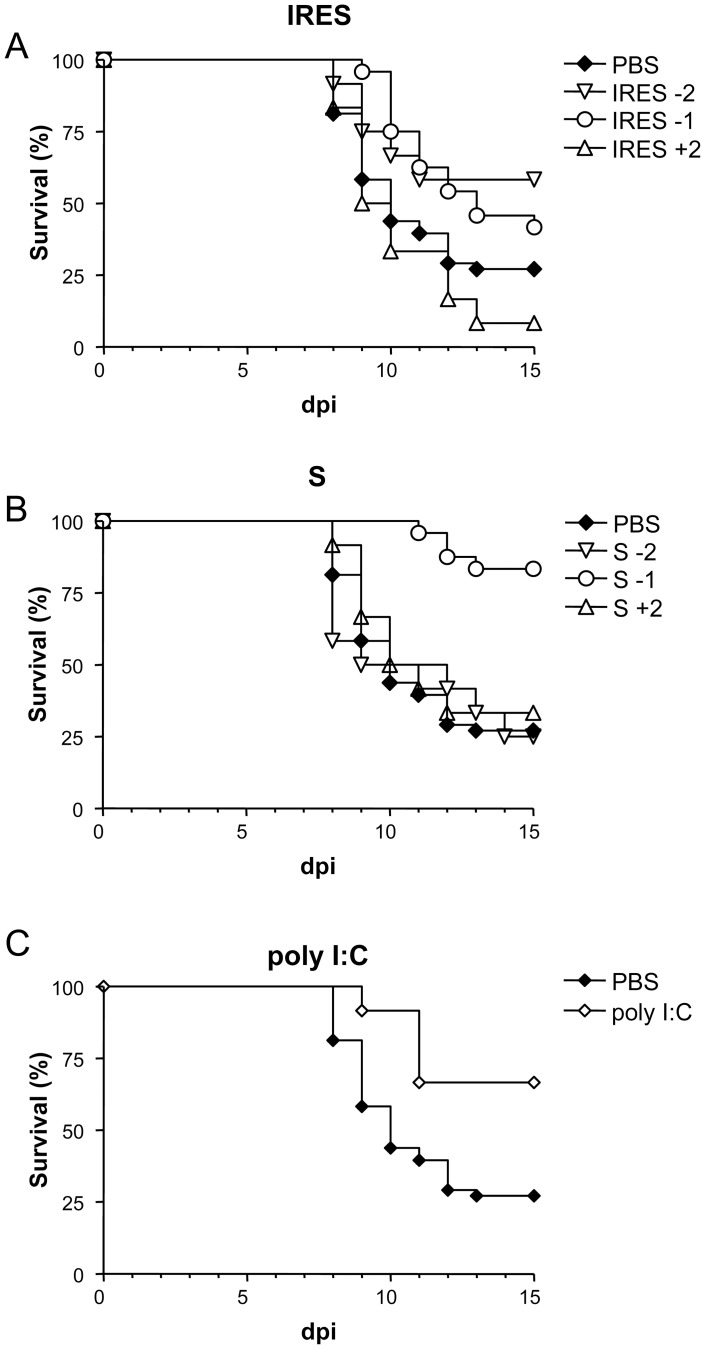
Survival rates of WNV infected adult mice inoculated with the different transcripts at various time intervals. Mice were i.p. inoculated with the corresponding transcripts, either the IRES (A), the S fragment (B), 1 (−1) and 2 (−2) days before and 2 (+2) days after i.p. infection with 10^5^ PFU/mouse of WNV, as described in Materials and Methods. Survival rates of mice inoculated with PBS alone or with poly (I:C) 24 h before infection are shown for comparison (C).

Since infection of adult mice is a more commonly used model for WNV studies, the activity of these RNAs against WNV infection was further analyze on them when administered before or after infection (Figure 3). Animals inoculated with the IRES 48 h or 24 h before i.p. infection with 10^5^ PFU of WNV showed higher survival rates (58.3%, p≤0.0741; and 41.7%, p≤0.0457, respectively) than those of the PBS-inoculated control group (22.2%), but these figures dropped to 8.3% when treated 48 h after infection (Fig. 3A). On the other hand, mice inoculated with S transcript presented survival rates of 25% and 33.3% when the transcripts were administered 48 h before or after infection, respectively, but the rate raised to 83.3% (p≤0.0001) when inoculated 24 h before infection (Fig. 3B). A 66.6% (p≤0.0135) survival rate was also observed in the group of mice treated with poly (I:C) 24 h before infection (Fig. 3C). Overall, MST values were higher when the transcripts where administered 24 h before infection than when inoculated 48 h before or after infection.

**Figure 4:**
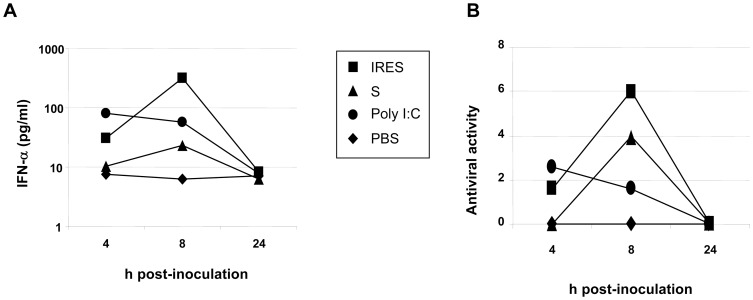
FMDV NCR transcripts trigger innate immunity in adult mice. Groups of adult mice were i.p. inoculated with 200 µg of the IRES, the S fragment, poly (I:C), or PBS. Levels of IFN-αwere measured for each group of mice in pools of sera collected at 4, 8 or 24 h, by ELISA (A), cut-off 25 pg/ml, and the antiviral activity was determined (B) on L929 cells, starting from 1∶1 sera dilution, as described in Materials and Methods.

Analysis of IFN-α levels in sera from inoculated adult animals (Fig. 4) showed that both transcripts elicited the highest levels at 8 h after inoculation and decreased to basal levels by 24 h (Fig. 4A). Similar results were observed when antiviral activity of these sera was tested in cell culture. In any case, IFN-α levels and antiviral activity were always higher in IRES-treated adult mice (Fig. 4B).

No specific anti-WNV antibodies were detected in the blood of treated mice euthanized early after infection (3–5 days), while high ELISA (median P/N = 8.5, range 7.5–11.5) and VNT (median 1/320, range 1/80 to <1/1280) titers were recorded in mice that survived to the infection by the end of the experiment (20 d.p.i.), regardless of the treatment received.

Over 55% (30/54) of the brains obtained from euthanized mice 3–5 d.p.i. resulted positive by RT-PCR for WNV-RNA with variable titers (average 1.2×10^5^ genomic equivalents/gram of tissue, range 2×10^2^–1.7×10^6^). These figures rose to 85.7% (12/14) when brains from mice that died of WND 8–9 d.p.i. were analyzed (average 3.3×10^6^ genomic equivalents/gram of tissue, range 4×10^2^–2.9×10^7^); however, the limited number of samples analyzed did not allow a proper comparison between the different groups.

## Discussion

The capability of non-infectious *in vitro* transcribed RNAs, mimicking structural domains of the NCRs of the picornavirus FMDV, to trigger the immune response in porcine cultured cells and mice [24], and to induce protection against challenge of suckling mice with FMDV homologous and heterologous strains have been reported [25]. Here, we have assessed the potential of two of these transcripts (the IRES and S fragments) to protect against infection with an important zoonotic unrelated virus, the flavivirus WNV, in both suckling and adult mice.

Protection against WNV infection was achieved in suckling mice after treatment with the two transcripts and the survival rates depended on the infecting viral dose, as previously shown for FMDV [25], and also on the infection route. Accordingly, while no protection was observed in any of the experimental groups in which mice were i.p. infected with a very high dose (10^7^ PFU/mouse) of WNV, a full protection was observed in animals i.c. infected with the lower dose administered (10 PFU/mouse), except in those of the control group that were inoculated with PBS alone whose showed mortality rates close to 100%, as previously reported in non-treated WNV-infected suckling mice [28].

Though the viral load inoculated by different species of mosquitoes varies (10^1^–10^6^ PFU), most of them inoculate around 10^2^ PFU directly into the blood when feeding on mice [35]. Our results indicate that, when 10^2^ PFU of WNV were used to i.c. infect suckling mice, relatively high protection rates were observed among those animals inoculated with the IRES, the S, or the mixture of both fragments, 24 h prior to the infection (64%, 44%, and 90.9%, respectively), which were lower than that of the control group (81.8%) inoculated with virus that had been previously incubated with a neutralizing anti-WNV polyclonal sera pool (n-sera). In any case, these survival rates were significantly higher than those recorded among the poly (I:C) (0%) and PBS (14.3%) inoculated control mice. On the other hand, MST in the PBS-inoculated mice group, although lower, was not markedly different than that of the remaining groups.

Even higher survival rates were observed in mice treated with the RNA transcripts 24 h before infection when the same infecting dose (10^2^ PFU/mouse) was administered by i.p. route instead of i.c. (82.6%, 96.4% and 100% for the IRES, the S fragment and the mixture of both, respectively). A full protection was also achieved in the control n-sera group. Again, these rates were significantly higher than those of the poly (I:C) (33.3%) and PBS (28%) groups. Despite that no marked differences in survival rates between treated and untreated mice infected with FMDV have been observed [25], a trend to lower MST was noticed in treated WNV-infected animals. This fact could be due to the different *in vivo* kinetics and pathogenicity of FMDV and WNV in newborn mice, as MST of 1.9 [25] and around 8.5 [28, and present report] days were respectively recorded in PBS-inoculated suckling mice.

The finding that i.p. treatment with the FMDV RNAs protected against direct infection into the brain of suckling mice indicates that the response induced was systemic. On the other hand, our results also showed that the route of viral inoculation is also important, as protection was higher after i.p. infection. WNV initially replicates at the inoculation site and then traffics to the lymph nodes and blood stream from where it reaches the spleen and kidneys and, finally, penetrates the CNS [11], where it is capable to directly infect neurons [21], [36]. Thus, in some cases, i.c. infection could probably allow enough viral replication in the brain before the level and efficacy of the IFN response induced by the transcripts has had time to stop disease progression.

When experiments were conducted using adult mice, similar results were observed, as 41.7% and 83.3% survival rates were recorded, respectively, among mice inoculated with the IRES or the S transcripts 24 h before infection with 10^5^ PFU of WNV, in comparison with the 22.2% survival rate observed in PBS-treated mice, which is similar to that previously documented in untreated adult mice [26], [36]–[38]. In this case, confirming previous data [23], protection was also obtained in adult mice after poly (I:C) administration (66.6%).

A previous study [25] showed that the high protection observed in suckling mice after FMDV challenge when the IRES (86%) and S (100%) transcripts were inoculated 24 h before FMDV infection, decreased when they were administered either 72 h (0% and 36%, respectively) or 48 h (56% and 50%, respectively) before challenge. Here, these results were confirmed, as little protection was observed in suckling mice upon inoculation with the transcripts 1 or 5 days after WNV infection when compared with that observed when administered 24 h before. The only noticeable exception (60% and 20% protection) was observed upon administration of the IRES 1 or 5 days after infection. These results remark that the potential prophylactic activity of these molecules has a quite narrow window. On the other hand, both transcripts protected adult mice when administered 1 day before WNV infection (42% and 83%), but some level of protection was also observed when administered 2 days before (58% and 25%, respectively) or after (8% and 33%, respectively) infection. The levels of IFN-α and antiviral activity in cultured cells detected in sera from treated adult mice supports these findings, since both RNAs induced a peak of IFN-α at 8 h post-inoculation that declined to basal levels 24 h later.

These observations are in accordance to those previously documented after administration of IFN, or IFN derivatives, whose activity is usually effective only when triggered previous or very close to the infection, because resistance to its effects once the infection has been established has been reported in cell culture, animal models and humans [8], [9], [22]. For instance, prophylactic treatment with IFN-α administered once daily for 7 days, starting 24 h before WNV infection, completely prevented death in adult mice, but treatment efficacy was strongly reduced (30% survival) when the drug administration was initiated 4 to 6 h before infection and maintained during 5 days [23]. Nevertheless, a case in which substantial beneficial effects were observed when IFN treatment was started in a patient with WN meningoencephalitis 3 weeks after disease presentation has been documented [10].

Adult mice that survived to the infection showed ELISA and VNT values similar to that previously described [28], but no differences were observed between the titers of mice treated with PBS or with the transcripts, suggesting that the level of viral replication induces a similar antibody response in all surviving mice.

As previously observed in WNV infected adult mice [26], [32], [36], [37], WNV-RNA was detected at quite variable levels in more than half of the mice euthanized 3–5 d.p.i, but no apparent differences were observed as a consequence of the inoculum (RNA transcripts or PBS) used or the inoculation schedule (before or after infection). However, it should be noted that only a small number of samples was analyzed in each group and, thus, further experiments are needed for a proper analysis.

Our results document that both RNA transcripts tested protect against WNV infection, and even though their activity window is narrow, they are helpful in activating the innate response against the infection. Their potential therapeutic use would benefit from further studies aimed to optimization of parameters such as delivery, dose range or route of administration, taking advantage of newly developed RNA-based technology [39], [40], The immunomodulatory effect of the IFN induced by these transcripts also suggests that they may be useful co-adjuvant molecules and, therefore, further analyses should be conducted to address this point. Nevertheless, the role played by the different cellular compounds involved in triggering the IFN response after administration of the RNAs used here remains to be elucidated.

In summary, our results extend the range of the *in vivo* antiviral activity raised by FMDV NCR RNA transcripts and remark their potential for prophylactic treatment against a variety of viruses.
